# Poly(ethylene glycol)s With a Single Cinnamaldehyde Acetal Unit for Fabricating Acid-Degradable Hydrogel

**DOI:** 10.3389/fchem.2020.00839

**Published:** 2020-09-15

**Authors:** Xinyue Zhao, Pengfei Shan, Haiwei Liu, Daai Li, Peihan Cai, Zhongyu Li, Zhihui Li

**Affiliations:** ^1^State Key Laboratory of Optometry & Vision Science, School of Optometry and Ophthalmology and Eye Hospital, Wenzhou Medical University, Wenzhou, China; ^2^College of Chemistry and Materials Engineering, Wenzhou University, Wenzhou, China; ^3^The Department of Neurology, The First Affiliated Hospital of Wenzhou Medical University, Wenzhou, China

**Keywords:** poly(ethylene glycol), hydrogel, cinnamaldehyde, acetal, acid-degradable

## Abstract

A synthetic route to prepare a poly(ethylene glycol) with a single cinnamaldehyde acetal unit in the polymer chain, was successfully established using a newly synthesized cinnamaldehyde acetal diethylene glycol (CADEG) as initiator. This HO-PEG(ca)-OH is non-toxic and would be degraded into a cinnamaldehyde and two PEG diols in acid environment. A whole polyethylene glycol based hydrogel was easily fabricated by thiol-ene “click” reaction in alkalescence aqueous solution using acrylate-PEG(ca)-acrylate and 4-arm PEG-SH as raw materials at room temperature. The gel time was dependent on the pH of the solution and its alkalinity can promote gel. The hydrogel can be degradable in acidic conditions and the stronger the acidity, the faster the degradation. This HO-PEG(ca)-OH also can be used in synthesis of cinnamaldehyde containing PEG derivatives, block copolymers or other acid degradable materials.

## Introduction

Poly(ethylene glycol) (PEG), a prominent synthetic polymer approved by the Food and Drug Administration (FDA) of the United States has been wildly used in material science, biology, cosmetics, drug excipient, typically in PEGylation of protein, drug, or gene owing to its peculiar properties, including non-toxic, chemically inert, extremely low immunogenicity, non-antigenicity, and well soluble in many organic solvents and water (Harris and Chess, 2003; Pasut and Veronese, 2012; Herzberger et al., 2016; Cabral et al., 2018). The polyether backbone structure of PEG provides excellent stability in aqueous solution, even in the circumstances of bloodstream (Herzberger et al., 2016). However, its non-degradable property is a key limitation for its application in therapeutics (Ulery et al., 2011). For example, to improve the circulation time of PEGylated proteins or to implement enhanced permeability and retention (EPR) effect of PEGylated drugs or nanoparticles, higher average molecular weight of PEG is needed. While with the increasing of the average molecular weight (*M*_*n*_), in particular when the *M*_*n*_ is higher than 40 kDa, the PEG will accumulate in the liver and result in organ damage due to its non-biodegradable property (Maeda et al., 2013). As a result, biodegradable PEGs with cleavable moieties in the backbone are in need and attract great interests. Many multiblock PEGs carrying cleavable moieties such as acetal (Tomlinson et al., 2003; Rickerby et al., 2005; Wang et al., 2011), imine or oxime (Collins et al., 2017), maleamic acid derivative (Su et al., 2018), ester (Wang et al., 2004), and disulfide (Braunova et al., 2007) in the backbone were synthesized successfully using telechelic PEGs as raw material via step-growth mechanisms. For example, Reid et al. (2010) used Fenton's reagent to partially oxidize the ether bonds of PEG for generating hemiacetals at the PEG backbone. Lundberg et al. (2012) reported a method for synthesis of hydrolytic degradable PEG by incorporating methylene ethylene oxide units into the PEG backbone. Furthermore, to solve the lack of well-define property of PEGs synthesized by aforementioned strategies, Dingels et al. (2013) and Pohlit et al. (2017) recently developed new methodologies of the implementation of acetal or ketal containing initiators for the anionic ring-opening polymerization of ethylene oxide to fabricate acid labile well-defined PEGs directly.

Cinnamaldehye, a major component in cinnamon which is approved by FDA for use in food, has been wildly used as a flavoring agent in food, cosmetics, and perfumes (Cocchiara et al., 2005; Ashakirin et al., 2017; Zhu et al., 2017). Cinnamaldehyde also has potential applications in pharmaceutics. For example, cinnamaldehyde has antimicrobial property due to its inhibition of bacterial or yeast growth (Lopez et al., 2007). Cinnamaldehyde blocks the formation of Tau protein aggregation into neurofibrillary tangles, which is a major pathology in Alzheimer's Disease (George et al., 2013). Cinnamaldehyde and its analogs are potent inducers of apoptosis via reactive oxygen species (ROS)-mediated mitochondrial permeability transition in various human cancer cells (Ka et al., 2003). However, its poor stability and solubility in aqueous solution, lack of specificity toward diseased tissue, and short half-life limit its use in clinical applications (Kim et al., 2013; Zhu et al., 2017).

In this report, a novel cinnamaldehyde acetal-containing initiator, namely (3-phenylprop-2-ene-1, 1-diyl) bis(oxy)diethanol or cinnamaldehyde acetal diethylene glycol (CADEG), and its corresponding HO-PEG(ca)-OH, a poly(ethylene glycol) with a single cinnamaldehyde acetal (ca) unit in the polymer chain are described. Acrylate-PEG-acrylate was synthesized as a model of PEG derivatives by post modification of PEG diol with acryloyl chloride. A whole polyethylene glycol based hydrogel was easily fabricated by thiol-ene “click” reaction in alkalescence aqueous solution using acrylate-PEG(ca)-acrylate and 4-arm PEG-SH as raw materials at room temperature. And the hydrogel would be degradable in acidic conditions. Furthermore, a triblock amphiphilic copolymer HO-PCL-b-PEG(ca)-b-PCL-OH was also successfully demonstrated by ring-opening polymerization of ε-caprolactone (ε-CL) using HO-PEG(ca)-OH as initiator.

## Materials and Methods

### Materials

All starting compounds were used as received without additional purification except for those specified. Chemicals were purchased from Aladdin (Shanghai) unless otherwise indicated. THF (99%) was refluxed over sodium wire and distilled from sodium naphthalenide solution. DMSO was distilled over CaH_2_ under reduced pressure just before use. Ethylene oxide (EO, 99.7%) was purchased from Xiapu Chemical Company and was distilled over CaH_2_ just before use. Diphenylmethylpotassium (DPMK) was prepared as described elsewhere (Li et al., 2006). 2-acetoxyethyl vinyl ether (compound **1**) was prepared according to a previous protocol (Greenland et al., 2010).

### Methods

^1^H NMR spectra were obtained on a DMX 400 MHz spectrometer with tetramethylsilane (TMS) as the internal standard and CDCl_3_ as the solvent. Size exclusion chromatography (SEC) was performed in 0.1 M NaNO_3_ at 40°C with an elution rate of 1.0 mL/min on a Waters HPLC system with a G1310A pump and a 2414 refractive index (RI) detector, Ultrahydrogel 250 (Waters), and Ultrahydrogel 1000 (Waters) columns in series were calibrated by polyethylene glycol standard. For triblock copolymer PCL-b-PEG-b-PCL, its SEC test was performed in THF at 35°C with an elution rate of 1 ml/min through a 7.8 × 300 mm column (with guard column) and a 2414 RI detector with polystyrene (PS) standard. MALDI-TOF MS spectra were recorded using Bruker REFLEX III. α-Cyano-4-hydroxycinnamic acid (CHCA) was used as the matrix for the ionization operated in the reflection mode. The element analysis of the product was recorded on an Elementar *Vario EL*-*III* (Germany). Rheological measurements were performed on a DHR-2 controlled stress rheometer (TA instruments, USA) with a parallel plate geometry (diameter = 8 mm). The strain was kept within the limits of the linear viscoelastic regime at a temperature of 25°C. The rubber-elastic plateau was determined from the frequency independent regime of the storage modulus.

### Synthesis of (3,3-Dimethoxyprop-1-enyl)benzene (2)


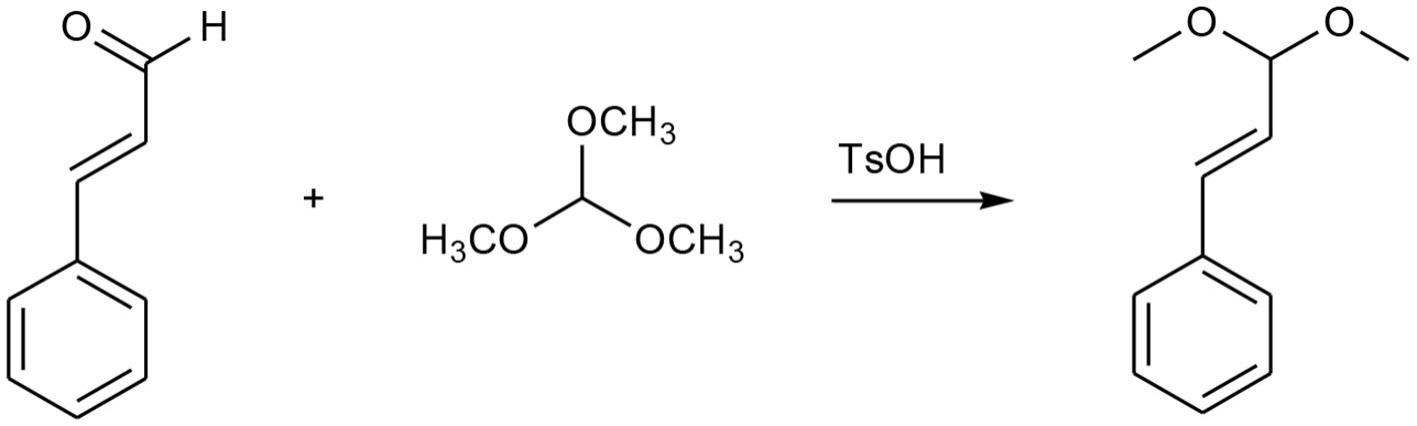


To a solution of cinnamaldehyde (6.6 g, 0.05 mol) in 60 mL dried methanol, trimethyl orthoformate 20.0 g (0.188 mol) and 30 mg (0.015 mmol) *p*-toluenesulfonic acid monohydrate was added with a stirrer bar under nitrogen atmosphere. The reaction mixture was refluxed at 70°C for 6 h then cooled to room temperature. The resulting crude reaction mixture was concentrated under reduced pressure. The crude product was further purified by column chromatography with EtOAc/petroleum spirit (4/1, v/v) as eluent to produce a yellowy liquid (3,3-dimethoxyprop-1-enyl)benzene (compound **2**) (7.6 g, yield: 85.4%). ^1^H NMR (500 MHz, CDCl_3_): δ [ppm] 7.47 – 7.19 (m, 5H), 6.72 (d, J = 16.1 Hz, 1H), 6.15 (dd, J = 16.1, 3.6 Hz, 1H), 4.96 (d, 1H), 3.36 (s, 6H).

### Synthesis of (3-Phenylprop-2-ene-1, 1-diyl) Bis(oxy)bis(ethnane-2,1-diyl) Diacetate (3)

To a solution of compound **2** (4.0 g, 0.022 mol) in 60 mL dry chloroform, 7.0 g 2-acetoxyethyl vinyl ether (compound **1**), 0.05 g hydroquinone (inhibitor), and 0.02 g (0.01 mmol) p-toluenesulfonic acid monohydrate were added at room temperature. The reaction mixture was refluxed at 65°C for 6 h then was concentrated under reduced pressure. The crude product was further purified by column chromatography with EtOAc/petroleum spirit (6/1, v/v) as eluent to produce a yellowy liquid (3-phenylprop-2-ene-1, 1-diyl) bis(oxy)bis(ethnane-2,1-diyl) diacetate (compound **3**) (5.61 g, yield: 77.6%). ^1^H NMR (500 MHz, CDCl_3_) δ [ppm] 7.47 – 7.19 (m, 5H), 6.78 (d, J = 16.1 Hz, 1H), 6.15 (dd, J = 16.1, 3.6 Hz, 1H), 4.96–4.86 (d, 1H), 4.20 (m, 2H, AcO*CH*_2_-), 3.68 (m, 2H, AcOCH_2_-*CH*_2_-), 2.05 (s, 3H, *CH*_3_COO-).

### Synthesis of Cinnamaldehyde Acetal Diethylene Glycol (CADEG) (4)

Potassium hydroxide (1.00 g, 17.8 mmol) and compound **3** (1.00 g, 3.1 mmol) were stirred under reflux in a solution of ethanol (10 mL) and water (0.5 mL) for 3 h. After cooling, brine was added and the solution was extracted with DCM three times. After drying over sodium sulfate the organic phase was evaporated to small volume. Pure product innamaldehyde acetal diethylene glycol (CADEG, compound **4**) (0.71 mg, 96.1%) was obtained by column chromatography (eluent: EtOAc/petroleum spirit = 2/1, v/v) over silica.^1^H NMR (500 MHz, CDCl_3_) δ [ppm] 7.47–7.19 (m, 5H), 6.78 (d, J = 16.1 Hz, 1H), 6.15 (dd, J = 16.1, 3.6 Hz, 1H), 4.96 (d, 1H), 3.70 (m, 2H, OH-*CH*2*-*CH_2_-), 3.75–3.50 (m, 4H, OH-*CH*2*-CH*_2_-). Elemental analysis: Calculated: C 65.53, H 7.61; Found: C 65.48, H 7.58.

### Synthesis of HO-PEG(ca)-OH With a Single Cinnamaldehyde Acetal Unit Amid the Chain (5)

A 150 mL stainless steel kettle was vacuumed at 90°C for 24 h and cooled to room temperature and then to 0°C under water ice bath. In the anhydrous CADEG (compound **4)** (2.46 g, 0.01 mol) dissolved in 40 mL of mixed solvents of DMSO and THF (v/v: 3/2), a solution of DPMK in THF (5.2 mL, 0.6 M solution) was slowly introduced. The orange-red color of DPMK was changed to yellow when the alkoxide was formed. Afterward, the homogeneous initiator solution obtained was introduced into the cooled kettle by a syringe, then ethylene oxide (EO) (37.4 g, 0.85 mol) was added. After the solution was stirred at 60°C for 24 h, the polymerization was terminated by adding of a few drops of acidified methanol (0.1 N HCl in ethanol). Then all the solvents were removed by reduced distillation. The crude product was dissolved in CH_2_Cl_2_, filtered, and dried over anhydrous MgSO_4_, then precipitated in diethyl ether. The white powder HO-PEG(ca)-OH was obtained in the yield of 95% and characterized by ^1^H NMR and SEC with refractive index detector and MALDI-TOF MS.

### Synthesis of Acrylate-PEG(ca)-acrylate (6)

One gram dry HO-PEG(ca)-OH (0.26 mmol) and TEA (0.06 g, 0.59 mmol) were dissolved in 20 mL of dry DCM and cooled to 0°C in an ice-bath. To the above solution, acryloyl chloride (1.81 g, 0.020 mol) was added dropwise over 30 min. The reaction was allowed to slowly warm up to room temperature and then stirred overnight. The reaction mixture was neutralized by 0.1 N HCl and washed with brine (2 × 15 mL), dried over MgSO_4_, filtered, the solvent removed on a rotary evaporator, then precipitated in diethyl ether. The white powder acrylate-PEG(ca)-acrylate (compound **6**) was obtained in the yield of 92% and characterized by ^1^H NMR and SEC with refractive index detector. GPC: *M*_*n*_= 4.13 × 10^3^ g/mol, *M*_*w*_*/M*_*n*_= 1.09.

### Synthesis of Triblock Copolymer HO-PCL-b-PEG(ca)-b-PCL-OH (7) From HO-PEG(ca)-OH

The dried HO-PEG(ca)-OH (1.95 g, 0.50 mmol) was weighed into a dry flask and 3.90 g (34.2 mmol) ε-caprolactone was subsequently added. The reaction mixture was stirred for 5 min at 130°C in a preheated oil bath before the catalyst (stannous octoate, 1 drop) was added and the polymerization was performed for 12 h. The resulting viscous was cooled to room temperature. The crude polymer was dissolved in dichloromethane and precipitated into diethyl ether twice to afford a white powder (5.46 g, yield = 93.3%). *M*_*n*_ (^1^H NMR) = 11.2 × 10^3^ g/mol; SEC (polysyrene standard): *M*_*n*_= 10.2 × 10^3^ g/mol, *M*_*w*_*/M*_*n*_= 1.18.

### Synthesis of 4-arm PEG-OH (8) and 4-arm PEG-SH (9)

A 150 mL stainless steel kettle was vacuumed at 90°C for 24 h and cooled to room temperature and then to 0°C under water ice bath. In the anhydrous pentaerythritol (1.36 g, 0.01 mol) dissolved in 60 mL of mixed solvents of DMSO and THF (v/v: 3/2), a solution of DPMK in THF (5.2 mL, 0.6 M solution) was slowly introduced. The orange-red color of DPMK was changed to yellow when the alkoxide was formed. Afterward, the homogeneous initiator solution obtained was introduced into the cooled kettle by a syringe, then ethylene oxide (EO) (38.6 g, 0.88 mol) was added. After the solution was stirred at 60°C for 24 h, the polymerization was terminated by adding of a few drops of acidified methanol (0.1 N HCl in ethanol). Then all the solvents were removed by reduced distillation. The crude product was dissolved in CH_2_Cl_2_, filtered, and dried over anhydrous MgSO_4_, then precipitated in diethyl ether. The white powder 4-arm PEG-OH was obtained in the yield of 92%. ^1^H NMR (500 MHz, CDCl_3_) δ [ppm]: 3.43 [s, 8H, C(*CH2*)_4_-], 3.48-3.92 [-(CH_2_CH_2_O)_n_-]. *M*_*n*_ (^1^H NMR) = 3.97 × 10^3^ g/mol; SEC*: M*_*n*_= 3.92 × 10^3^ g/mol, M_w_/M_n_ = 1.17.

4-arm PEG-SH was synthesized by the esterification between 4-arm PEG-OH and 3-mercaptopropionic acid. Typically, 5 g of 4-arm PEG-OH (*M*_*n*_ = 3.92 × 10^3^ g/mol, 1.28 mmol) was dissolved in 10 mL toluene, then 2.16 g 3-mercaptopropionic acid (MPA) (20.0 mmol) along with HfCl_4_ ·2THF (5 mg, 0.01 mmol) were added and stirred at 50°C under nitrogen. The reaction flask was equipped with an azeotropic distillation apparatus and the mixture was refluxed at 110°C under nitrogen for 24 h. Toluene was removed under reduced pressure. The crude product was dissolved in CH_2_Cl_2_, dried over anhydrous MgSO_4_, filtered, and concentrated. The polymer was precipitated from an excess volume of diethyl ether twice to afford a white powder (4.7 g, yield = 85%). *M*_*n*_ (^1^H NMR) = 4.21 × 10^3^ g/mol; SEC: *M*_*n*_= 4.15 × 10^3^ g/mol, *M*_*w*_*/M*_*n*_= 1.18.

### Degradation of HO-PEG(ca)-OH in Acid


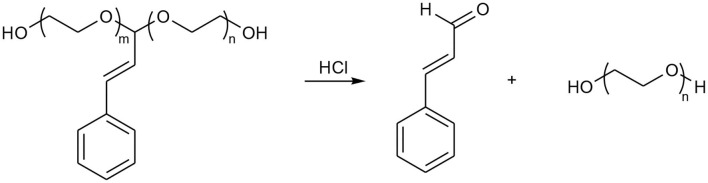


1.0 g HO-PEG(ca)-OH with a single cinnamaldehyde acetal unit was dissolved in two buffer solutions at pH 5.0 and 7.4, respectively, and incubated at 37°C for a set time. After hydrolysis, the solution was extracted by dichloromethane three times. After the solvents were removed under reduced pressure, the residue was dissolved in CH_2_Cl_2_, dried over anhydrous MgSO_4_, filtered, and concentrated. The polymer was precipitated from an excess volume of diethyl ether twice to afford a white powder (0.85 g, yield = 85%). SEC: *M*_n_ = 1.90 × 10^3^ g/mol, *M*_*w*_*/M*_*n*_= 1.10.

### Fabrication of Hydrogel by Thiol-ene “Click” Reaction Between Acrylate-PEG(ca)-acrylate and 4-arm PEG-SH

Typically, 2 g acrylate-PEG(ca)-acrylate (*M*_*n*_= 4.13 × 10^3^ g/mol) was dissolved in 3 mL pH = 8.0 PBS buffer to form A solution and 1 g 4-arm PEG-SH (*M*_*n*_= 4.15 × 10^3^ g/mol) was dissolved in 4 mL pH = 8.0 PBS buffer to form B solution. Then took the same volume of A and B solutions into a vial and vigorously shaking to mix them well. The hydrogel was formed for a period of time according to different pH of PBS buffer or polymer content.

### Fabrication of PEG Based Soft Contact Lens

Typically, 2 g acrylate-PEG(ca)-acrylate (*M*_*n*_= 4.13 × 10^3^ g/mol) was dissolved in 8 mL pH=8.0 PBS buffer to form A solution and 1 g 4-arm PEG-SH (*M*_*n*_= 4.15 × 10^3^ g/mol) was dissolved in 4 mL pH = 8.0 PBS buffer to form B solution. Then took the same volume of A and B solutions into a vial and vigorously shaking to mix them well. Then quickly draw the solution into the mold and place it for a while.

### Cell Viability Assay by the MTS Assay

Human NIH 3T3 cells were purchase from the American Type Culture Collection (ATCC) and cultured in Dulbecco's modified Eagle's medium (DMEM) supplemented with 10% fetal calf serum (FBS) at 37°C in a 5% CO_2_ humidified atmosphere. The cytotoxicity of the polymer was detected by MTS assay. NIH 3T3 cells were seeded into 96-well plates at a density of 5.0 × 10^3^ per well. After incubating for 24 h, various concentrations (0.00001, 0.0001, 0.001, 0.01, 0.1, and 1 mg/mL, respectively) of HO-PEG(ca)-OH (*M*_*n*_ = 3.88 × 10^3^ g/mol) and HO-PEG-OH (*M*_*n*_= 1.90 × 10^3^ g/mol) which was recovered from the hydrolytic residue of HO-PEG(ca)-OH in acid, were added and incubated for 24, 48, and 72 h at 37°C. Cells were treated by the MTS-based CellTiter 96® aqueous one solution cell proliferation assay reagent (Promega Corp., WI, USA) at 37°C for 3 h according to the manufacture's protocol. The absorbance was read at a wavelength of 490 nm with a SpectraMax Plus 384 Microplate Reader (Molecular Devices, Sunnyvale, CA, USA) and the cell viability was calculated.

### Statistical Analysis

All experiments in this investigation were performed at least three times and the data were expressed as mean ± standard deviation (SD). Statistical analysis was performed through *t*-test and analysis of variance (ANOVA).

## Results and Discussion

### Synthesis of Initiator

**Scheme 1:**
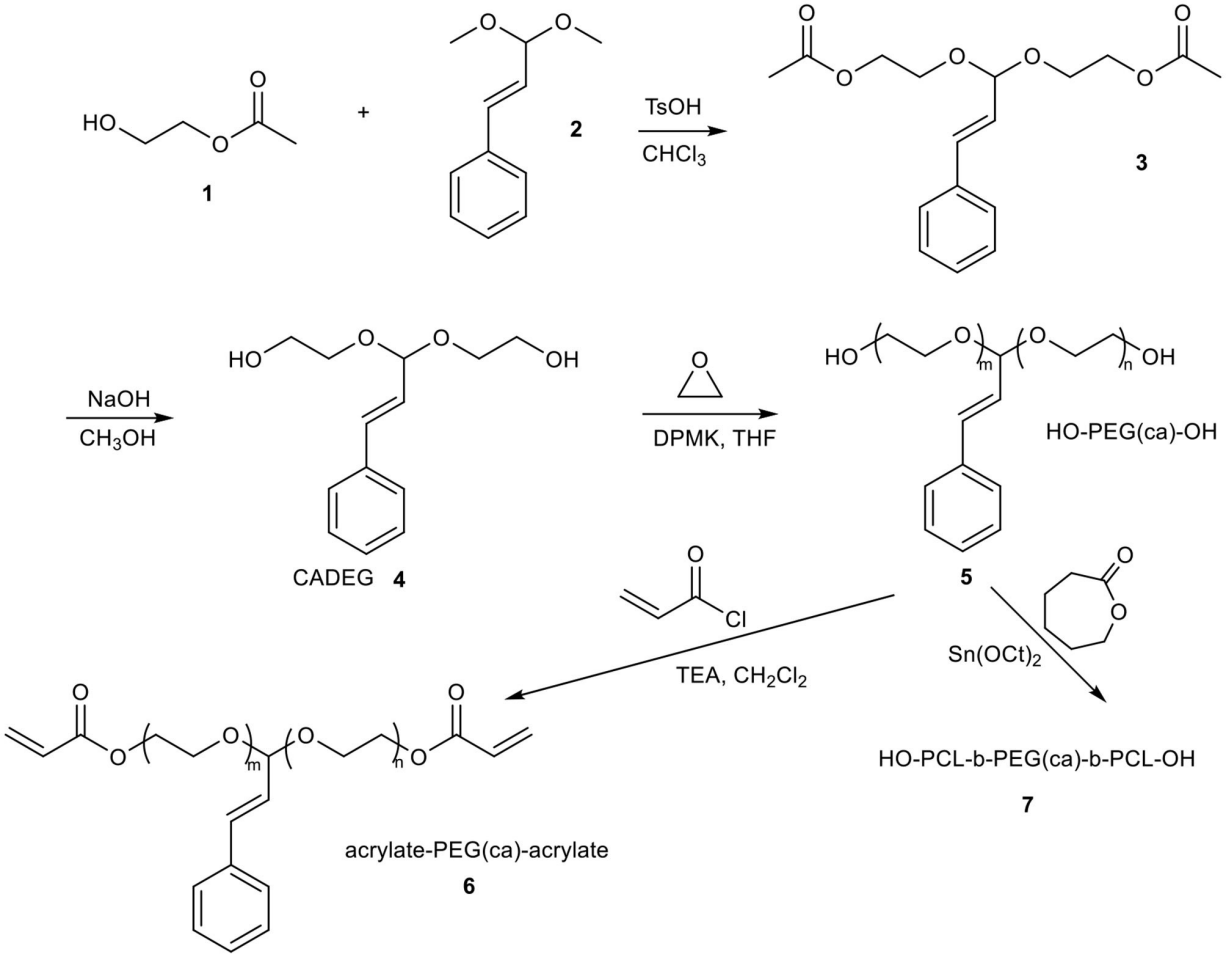
Synthesis of HO-PEG(ca)-OH and its derivatives.

**Figure 1:**
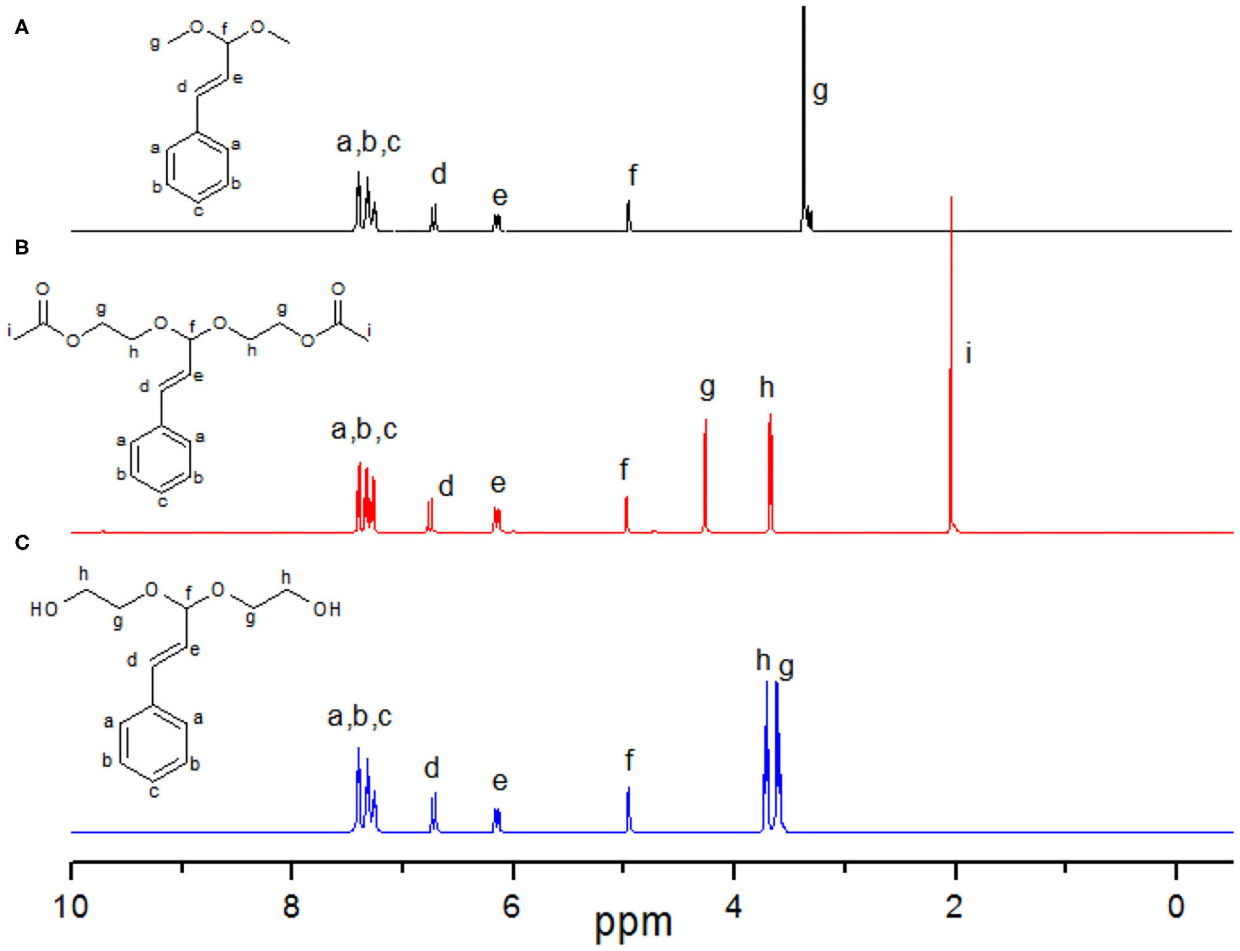
^1^H NMR spectra of initiator CADEG **(C)** and its two precursors **(A,B)** in CDCl_3_.

Acetal or ketal containing initiator or monomer was broadly used in anionic polymerization of epoxides for synthesis of acid-labile linear or hyperbranched polymers owing to the stability and tolerating the harsh conditions of the anionic polymerization of acetal or ketal moieties (Feng et al., 2011; Shenoi et al., 2012; Tonhauser et al., 2012; Dingels et al., 2013; Pohlit et al., 2017). These polymers can generate acetaldehyde or acetone from polyacetal or polyketal, respectively, when they were hydrolyzed in acid niches, for example, in solid tumor (Chen et al., 2010; Binauld and Stenzel, 2013). According to the literatures, the acetaldehyde (Garaycoechea et al., 2018) and acetone (Hewitt et al., 1980) are likely toxic and carcinogenic. Pursuing the safer and more rational degradable PEG is an important and challenge work for further designing of the next generation PEG for PEGylation or material science related to drug delivery. Drug based acetals seem to be good candidates as initiators or monomers for synthesis of acid labile polymers because the drug can be recovered when the polymers were hydrolyzed in acid circumstances, and no other toxic small molecules produced. With that in mind, the cinnamaldehyde acetal-containing initiator CADEG was designed and synthesized according to Scheme 1. The structures of CADEG and its precursors (compound 2-3) were characterized by ^1^H NMR (Figure 1) and elemental analysis (see Supporting Information). From the ^1^H NMR in Figure 1, all signals can be assigned to the corresponding protons verifying the structures of the initiator and its precursors.

### Synthesis of HO-PEG(ca)-OH and Its Derivatives

**Figure 2:**
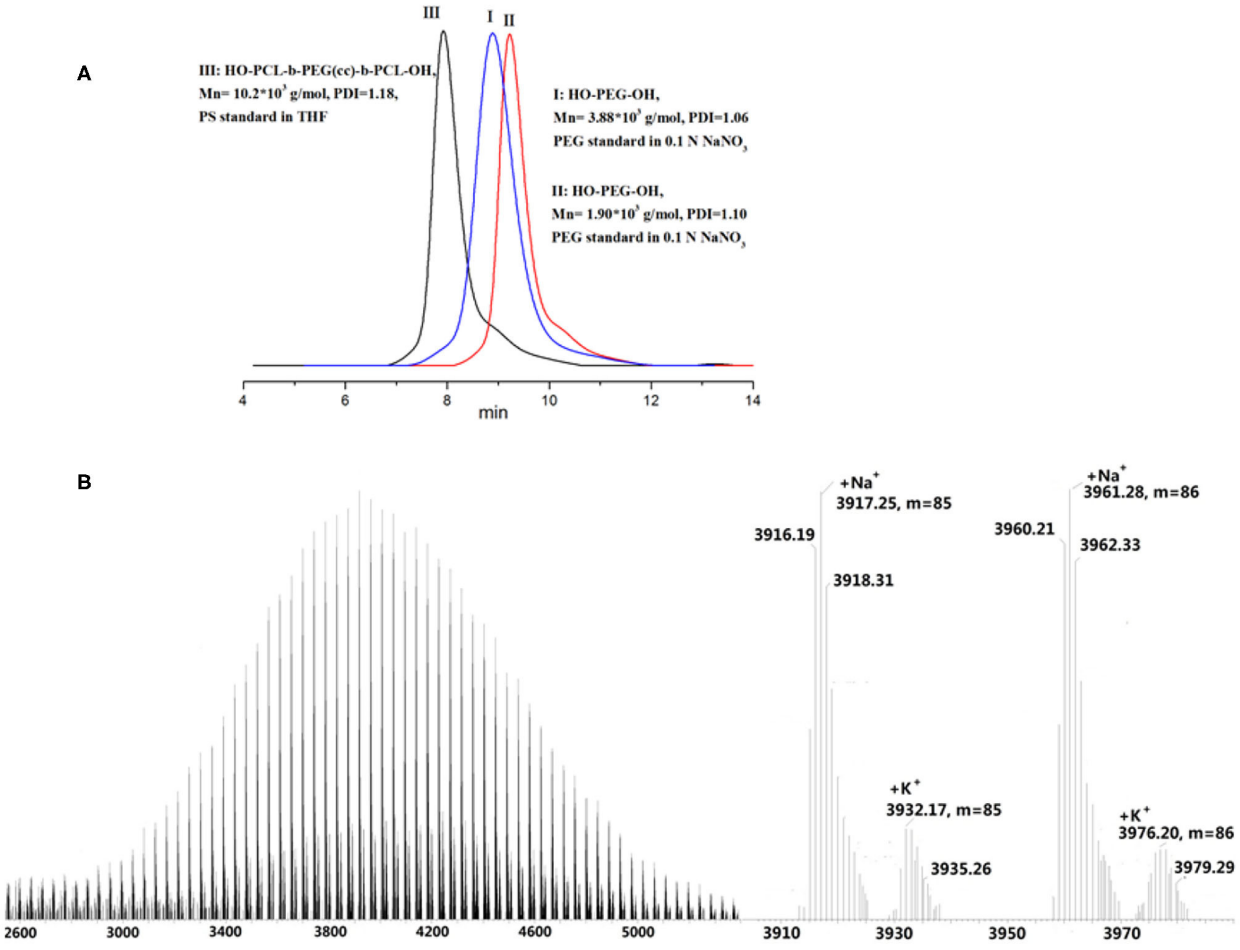
**(A)** SEC profile of HO-PEG(ca)-OH (I) (M_n_ = 3.88 × 10^3^ g/mol, PDI = 1.06), its degradable residue HO-PEG-OH (II) (M_n_ = 1.90 × 10^3^ g/mol, PDI = 1.10), and triblock copolymer HO-PCL-b-PEG(ca)-b-PCL-OH (III) (M_n_ = 10.2 × 10^3^ g/mol, PDI=1.18); **(B)** MALDI-TOF mass spectrum of HO-PEG(ca)-OH (M_n(SEC)_ = 3.88 × 10^3^ g/mol).

**Figure 3:**
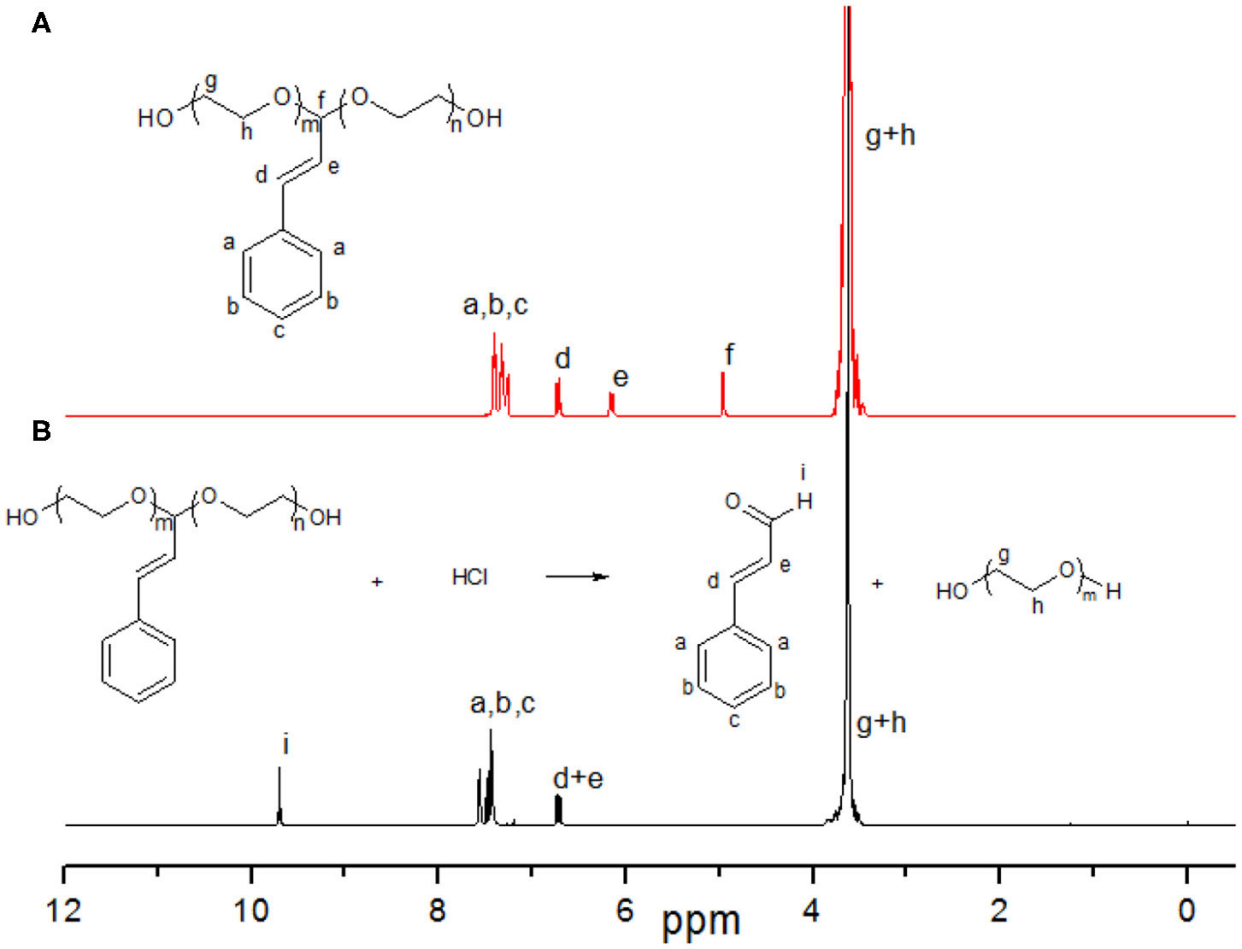
^1^H NMR spectra of HO-PEG(ca)-OH **(A)** and its degradable residue in acid **(B)** in CDCl_3_.

Poly(ethylene glycol)s were synthesized by the anionic polymerization of ethylene oxide (EO) in a stainless steel kettle using CADEG as initiator with diphenylmethylpotassium (DPMK) as a base^41^ for generating alkoxide initiators (Scheme 1). The polymerization was conducted in a DMSO/THF co-solvent system to ensure the complete solubility of cinnamaldehyde acetal based alkoxide and more importantly, a reduce rate of polymerization. The synthetic poly(ethylene glycol) with one cinnamaldehyde acetal unit was denoted as HO-PEG(ca)-OH. The synthetic condition and the results were listed in Table 1. The polymer was unimodal with an average molecular weight of 3.88 × 10^3^ g/mol and a narrow polydispersity of 1.06 according to SEC (Figure 2A). The structure of HO-PEG(ca)-OH was verified by ^1^H NMR spectrum (Figure 3A). The signals of the protons of the cinnamaldehyde acetal group are detected at δ (ppm) of δ 7.47–7.19 (m, 5H), 6.78 (d, 1H), 6.15 (dd, 1H), 4.96 (d, 1H). The number average molecular weight (M_n_) of the polymer was determined by ^1^H NMR spectrum using group analysis according to the following equation:

$$\begin{array}{l} M_{n}=44.05\times \left({\text{A}}_{\text{EO}}/4{\text{A}}_{\text{m}}\right)+\;150.17 \end{array}$$

**Table 1:** Results of anionic polymerization of ethylene oxide (EO) using CADEG as the initiator.

	**[EO]_**0**_/[CADEG]_**0**_**	**Time (h)**	**Yield (%)**	**10**^**−3**^** × M**_**n**_^**b**^		**10**^**3**^** × M**_**n**_^**e**^	**M_**w**_/M**_**n**_^**e**^
				**obsd^**c**^**	**calcd^**d**^**		
1a	41.5	24	92	1.95	2.0	1.96	1.09
2a	86.9	36	95	3.96	4.0	3.88	1.06
3a	177.7	48	93	7.91	8.0	7.86	1.08

a *Solvent: THF/DMSO = 3/2, Temperature: 60°C*.

b *M_n_ denotes number average molecular weight*.

c *Determined from the ^1^H NMR results*.

d *M_w_ denotes weight average molecular weight. ^d^Determined from the following equation: M_n_(calcd) = M_w_(EO)[EO]_0_/[CADEG]_0_ + M_w_(CADEG) = 44.05 [EO]_0_/[CADEG]_0_ + 238.28*.

e *Determined from the SEC results*.

Where A_EO_ and A_m_ are the peak areas of the sum of protons in the PEO main chain at δ = 3.45–3.80 ppm and methyne protons at δ = 4.96 ppm, respectively, 150.17 and 44.05 are the molecular weight of the cinnamaldehyde acetal moiety plus two protons of terminal hydroxyl group and the ethylene oxide (EO), respectively.

The *M*_*n*_ calculated by ^1^H NMR is 3.96 × 10^3^ g/mol, which is in close agreement with the measurement by SEC (M_n_ = 3.88 × 10^3^ g/mol) and by MALDI-TOF mass spectrum (*M*_*n*_ = 3.94 × 10^3^ g/mol, Figure 2B). The results of MALDI-TOF corroborate with the results of SEC, showing that the polymer is unimodal and has a low polydispersity of 1.04. The major series of the molecular masses of the product can be calculated by the following equation:

$$\begin{array}{l} M_{n}=\text{n}\cdot {\text{M}}_{\text{EO}}+\text{M}\left(\text{ca}\right)+{\text{M}}_{\text{w}}\left(\text{Metal ion}\right)=44.05\text{n}\\ \;+150.17+23.0\;\left(\text{sodium}\right) \end{array}$$

This result further confirmed that poly(ethylene glycol)s with a single cinnamaldehyde acetal unit has been successfully synthesized using CADEG as the initiator.

**Figure 4:**
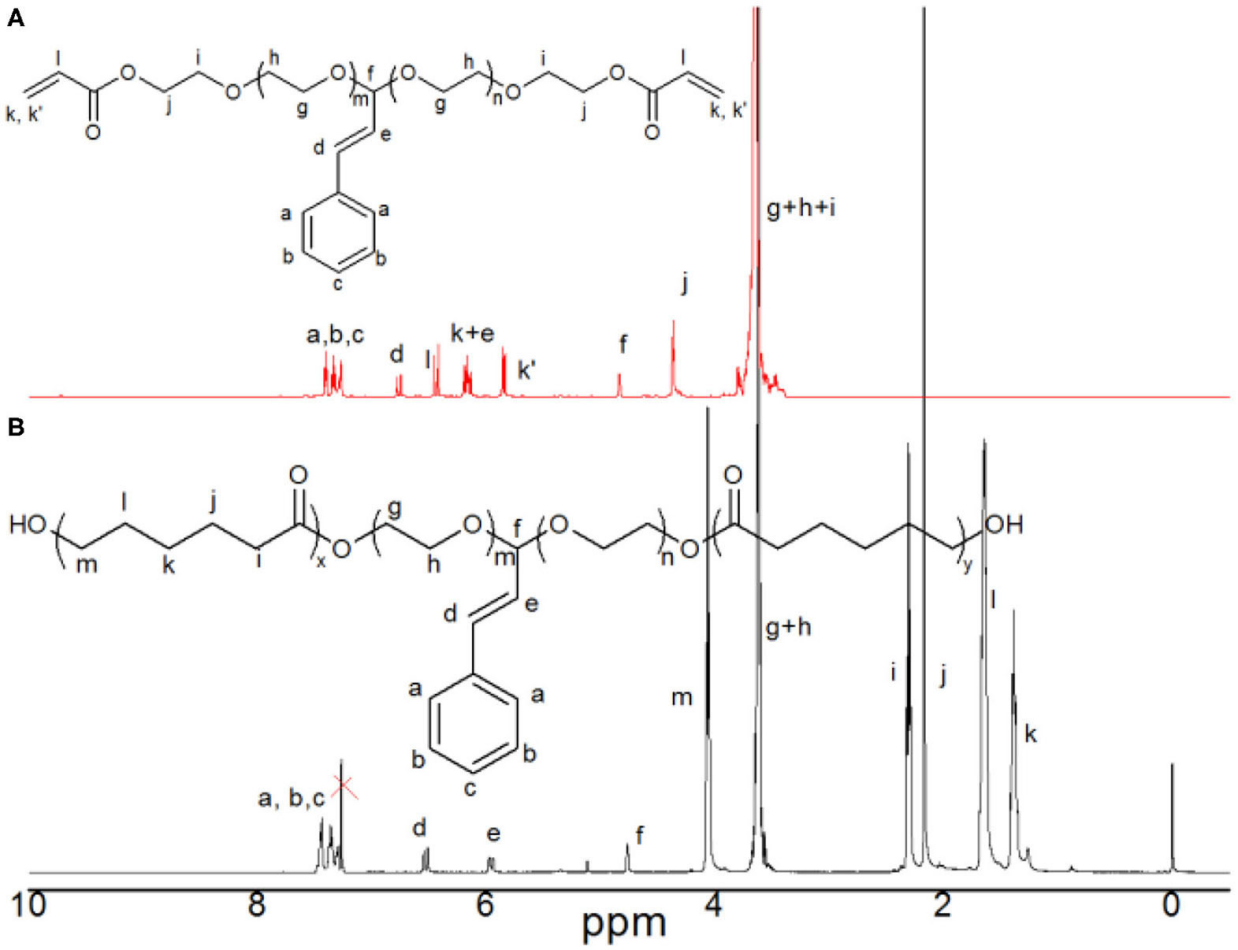
^1^H NMR spectra of acrylate-PEG(ca)-acrylate **(A)** and triblock copolymer HO-PCL-b-PEG(ca)-b-PCL-OH **(B)** in CDCl_3_.

According to our previous report (Li and Chau, 2009, 2011; Li et al., 2019), hydroxyl group can be modified into many other functional groups. Here, we took acrylate-PEG(ca)-acrylate as an example for post modification of HO-PEG(ca)-OH. The dried HO-PEG(ca)-OH was reacted with acryloyl chloride in anhydrous DCM at room temperature. The structure of the acrylate-PEG(ca)-acrylate was verified by ^1^H NMR in Figure 4A.

Furthermore, this HO-PEG(ca)-OH with two terminal hydroxyl group was used an macroinitiator for synthesis of an amphiphilic triblock copolymer HO-PCL-b-PEG(ca)-b-PCL-OH by ring opening polymerization of ε-caprolactone. The structure of the HO-PCL-b-PEG(ca)-b-PCL-OH was verified by ^1^H NMR in Figure 4B. The SEC results (Figure 2A) showed that it is unimodal, and the M_n_ of HO-PCL-b-PEG(ca)-b-PCL-OH is 10.2 kDa with 1.18 polydispersity. This amphiphilic triblock can be used in self-assemble for fabricating pH-responsive nanoparticles (Cabral et al., 2018).

### The Cytotoxicity of HO-PEG(ca)-OH

**Figure 5:**
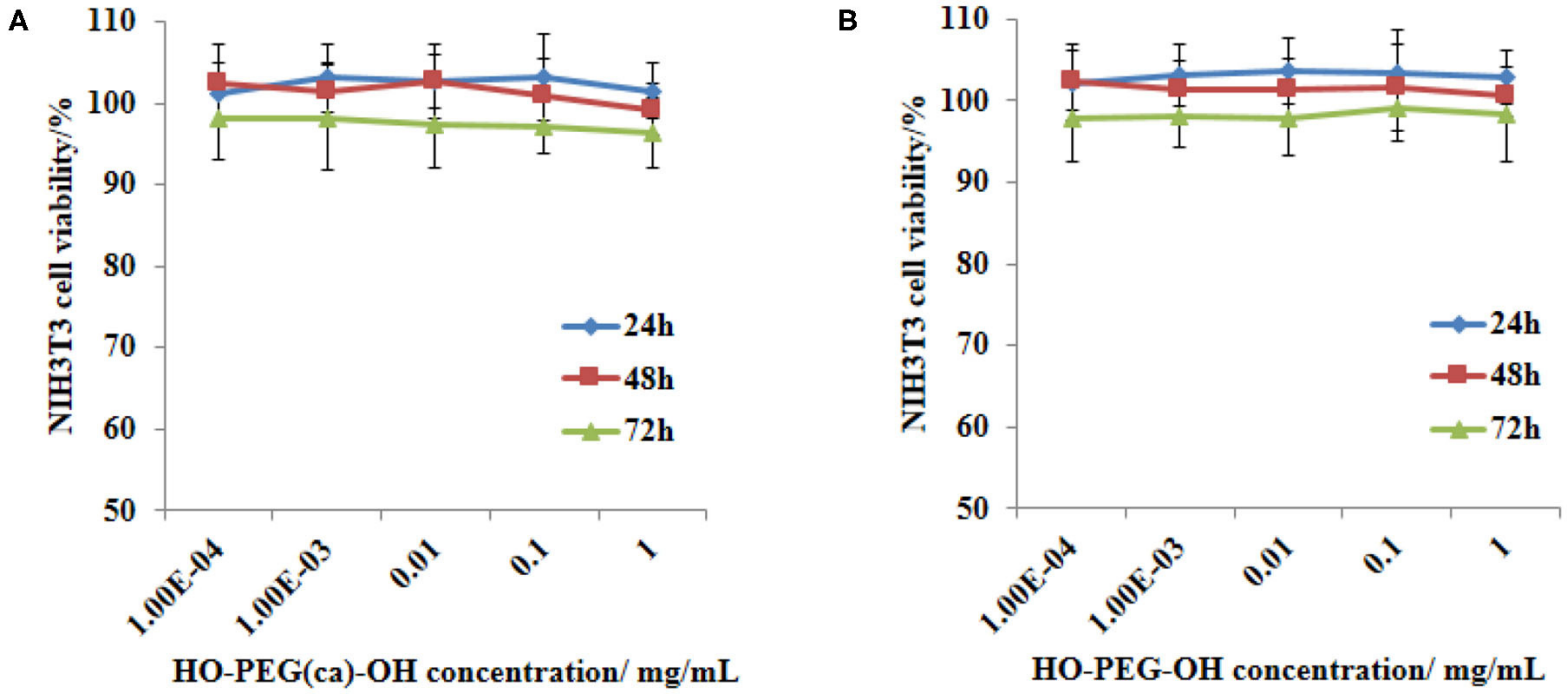
Cell viability data of HO-PEG(ca)-OH and its degradable residue by MTS assay. NIH3T3 cells were subjected to 0.00001, 0.0001, 0.001, 0.01, 0.1, 1 mg/mL of HO-PEG(ca)-OH (M_n_ = 3.88 × 10^3^ g/mol) and its degradable residue HO-PEG-OH (M_n_ = 1.90 × 10^3^ g/mol) for 24, 48, 72 h before undertaking a MTS assay. The results are shown as percent viability compared to the control with media only. Each sample was replicated 6 times.

The cytotoxicity of this HO-PEG(ca)-OH and its degradable product HO-PEG-OH were then determined for human fibroblast cells using MTS assay. No obvious loss in cell viability (Figure 5) was observed at all concentrations tested for this NIH3T3 cell line, indicated that both the HO-PEG(ca)-OH and its degradable product HO-PEG-OH are non-toxic. The cytotoxicity of cinnamaldehyde *in vitro* along with the anticancer ability of HO-PEG(ca)-OH based materials are currently under investigation in other research.

### Degradable of HO-PEG(ca)-OH in Acid Condition

**Figure 6:**
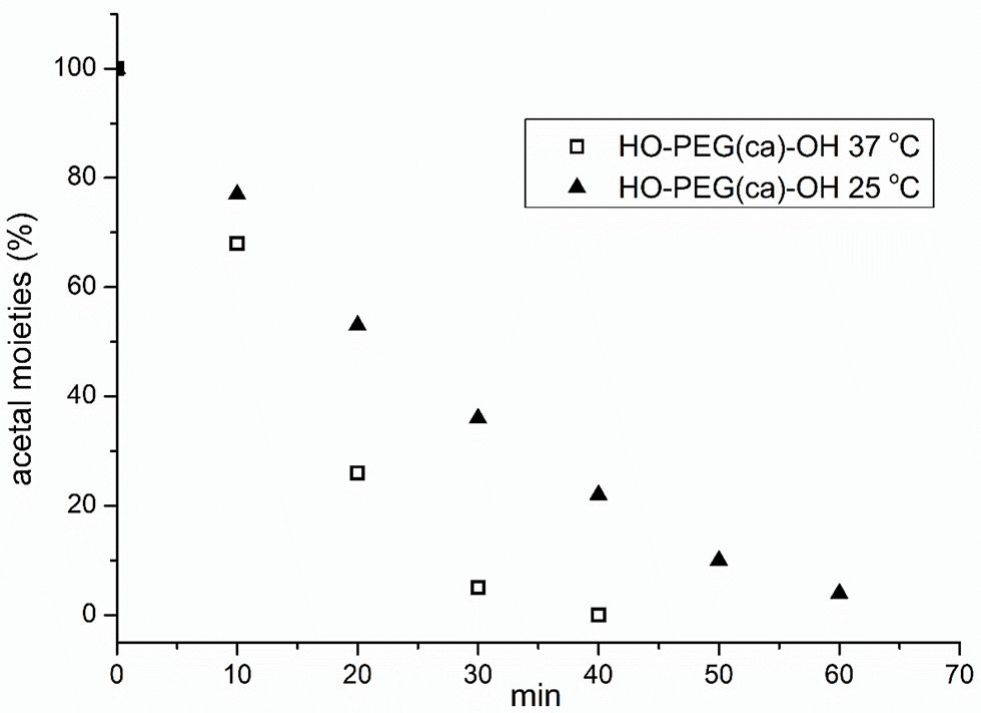
Decreasing acetal of the cinnamaldehyde acetal containing HO-PEG(ca)-OH at 25 and 37°C, respectively, in deuterated phosphate buffer at pH 5.0.

To investigate the degradation behavior of this PEG with a single cinnamaldehyde acetal unit, we compared the rate of hydrolysis in two buffer solutions at pH 5.0 and 7.4 at 37°C to mimic *in vivo* degradation conditions. After incubation 8 h, the residue was extracted by dichloromethane (DCM) and concentrated by rotary evaporation. The crude product was characterized by ^1^H NMR and SEC. The result of ^1^H NMR (Figure 3B) showed that in the case of hydrolysis in buffer at pH 5.0, the signals assigned to cinnamaldehyde acetal at δ 4.96 ppm (d, 1H, -O-CH(CHCH_2_-benzene)-O) disappeared completely and new signals assigned to cinnamaldehyde at δ 9.81 ppm -CHO appeared, indicating the complete degradation of HO-PEG(ca)-OH. This method also can be used to study the degradation kinetics of HO-PEG(ca)-OH. The polymers were incubated at 25 or 37°C in deuterated phosphate buffer at pH 5.0. After a certain time, the solution was neutralized immediately by 0.1 M NaOH and characterized by ^1^H NMR. As the degradation process going on, the signal of cinnamaldehyde acetal at δ 4.96 ppm will be weaker and weaker, and its integration can be used to calculate the degradation degree. The degradation kinetics was showed in Figure 6. Meanwhile, the decreasing of the average molecular weight of PEG from 3.94 to 1.90 kDa before and after hydrolysis with unimodal of PEG after hydrolysis by SEC results (Figure 2A) indicated that the cinnamaldehyde acetal group which is the cleavable point is on around the middle of the polymer chain. However, after incubation 3 days at pH 7.4, no obvious change of SEC profile with the absence of cinnamaldehyde signal by ^1^H NMR, verified that the HO-PEG(ca)-OH is stable in pH 7.4 PBS buffer solution. To further investigate storage conditions, samples were kept as dry powders at −18°C and in pH 7.4 PBS buffer solution at 4°C. No detectable degradation was found after two months when stored as a dry powder, and no appreciable degradation occurred within 3 days in pH 7.4 PBS buffer at 4°C. These results indicated that this HO-PEG(ca)-OH has the long term stability and the introduction of cinnamaldehyde acetal unit to PEG would enable the cinnamaldehyde delivery as well as the cleavability of PEG.

### Preparation of Hydrogels and Their Stability

**Figure 7:**
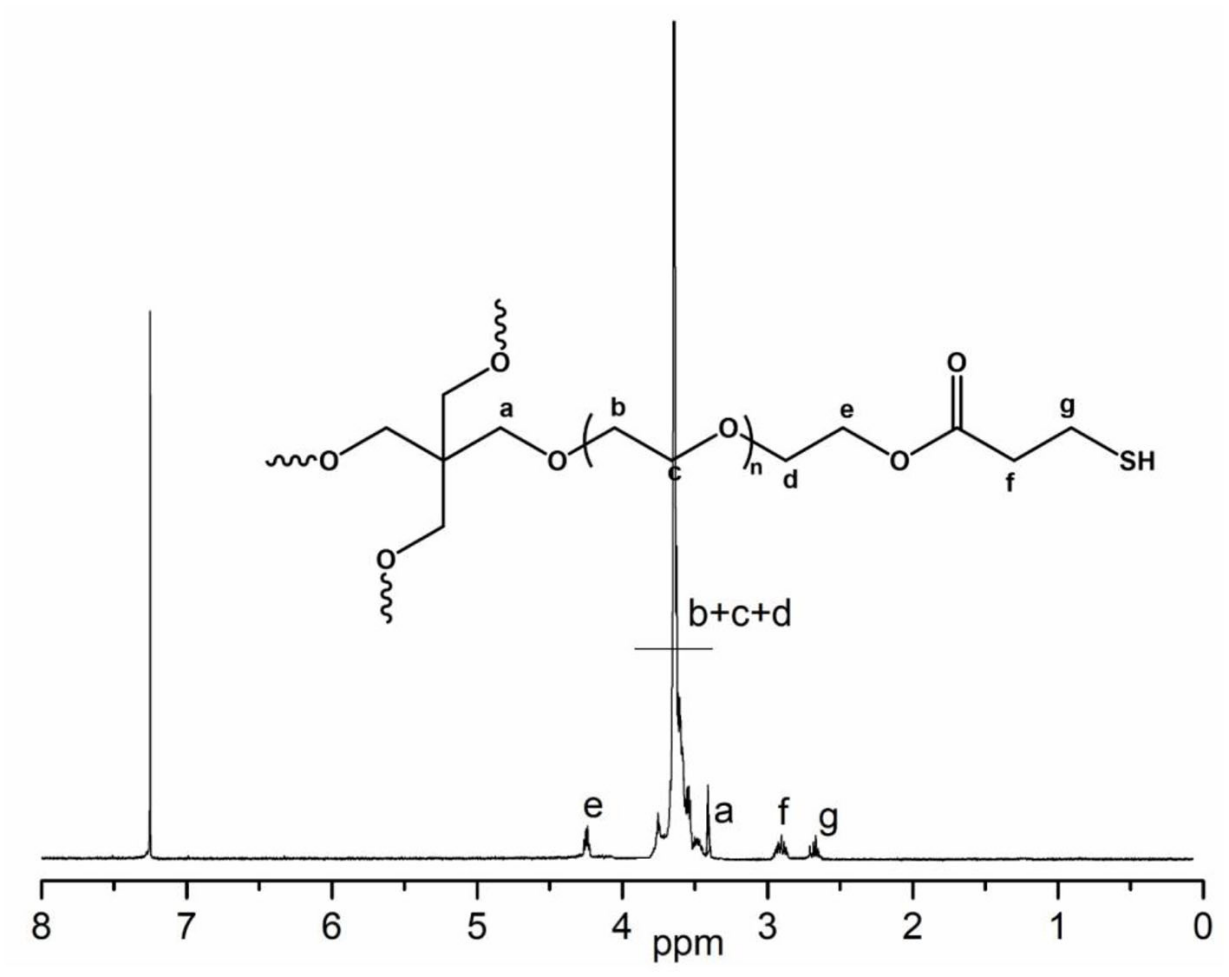
^1^H NMR of 4-arm PEG-SH in CDCl_3_.

**Scheme 2:**
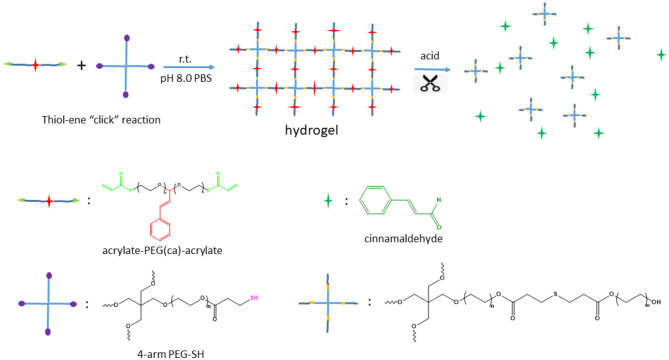
Fabrication and degradation of hydrogel.

**Figure 8:**
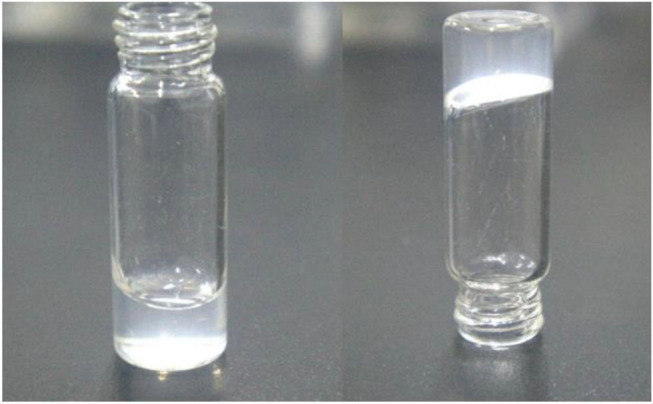
The optical images of the whole PEG based hydrogel (Right, gel H2) and its precursor aqueous solution (left).

Thiol-ene “click” reaction is a powerful tool for polymer synthesis, post modification and even in preparation of hydrogels (Hoyle and Bowman, 2010). Our synthesized acrylate-PEG(ca)-acrylate has the similar two acrylate terminal groups structure of PEG diacrylate (Lin et al., 2011), which was widely used in fabricating hydrogel, typically by photocrosslinking methods. Unfortunately, our acrylate-PEG(ca)-acrylate has a cinnamaldehyde group in the polymer chain, and the double bond on cinnamaldehyde group will also take part in photopolymerization of diacrylate or thiol-ene photo “click” reaction under UV light. So we chose the thiol-ene “click” reaction with alkaline as catalyst to fabricate hydrogel from acrylate-PEG(ca)-acrylate. To this end, we synthesized a 4-arm PEG-SH macromonomer and its structure was confirmed by ^1^H NMR (Figure 7). The process of fabricating hydrogel is easily as Scheme 2 showed. Typically, stoichiometric acrylate-PEG(ca)-acrylate and 4-arm PEG-SH (acrylate: SH = 1:1) were dissolved in alkalescence aqueous solution (such as PBS buffer), then mixed these two solutions together well, the hydrogel will be formed (Figure 8 and Video S1) for a period of time according to different polymer content and pH of solution (Table 2). The results in Table 2 suggest that the gel time was dependent on the pH of the solution, and the alkalinity or polymer content of solution can promote gel. As the hydrogel was crosslinking by polyethylene glycol with a cinnamaldehyde acetal group in the middle of polymer chain, the hydrogel must have the acid degradable property as the HO-PEG(ca)-OH mention above. To study the acid degradable property of hydrogel, we dip the hydrogel (H2) in PBS buffer with pH 5.0, 6.0, 7.4, and 8.0, respectively. The results showed that the hydrogel will be completely degraded in pH 5.0 PBS buffer after 16 h, and 36 h for pH 6.0 PBS buffer. No detectable degradation was found after 72 h for PBS buffer with pH 7.4 and 8.0.

**Table 2:** Gel time under different pH by inverted vial and visual check.

**Gel**	**Polymer content (%)**	**pH of solution**	**Gel time (s)**
1	15	7.4	No gel
2	15	7.7	720
3	15	8.0	180
4	15	8.2	25
5	15	8.4	7
H1	20	8.0	125
H2	15	8.0	180
H3	10	8.0	287

**Figure 9:**
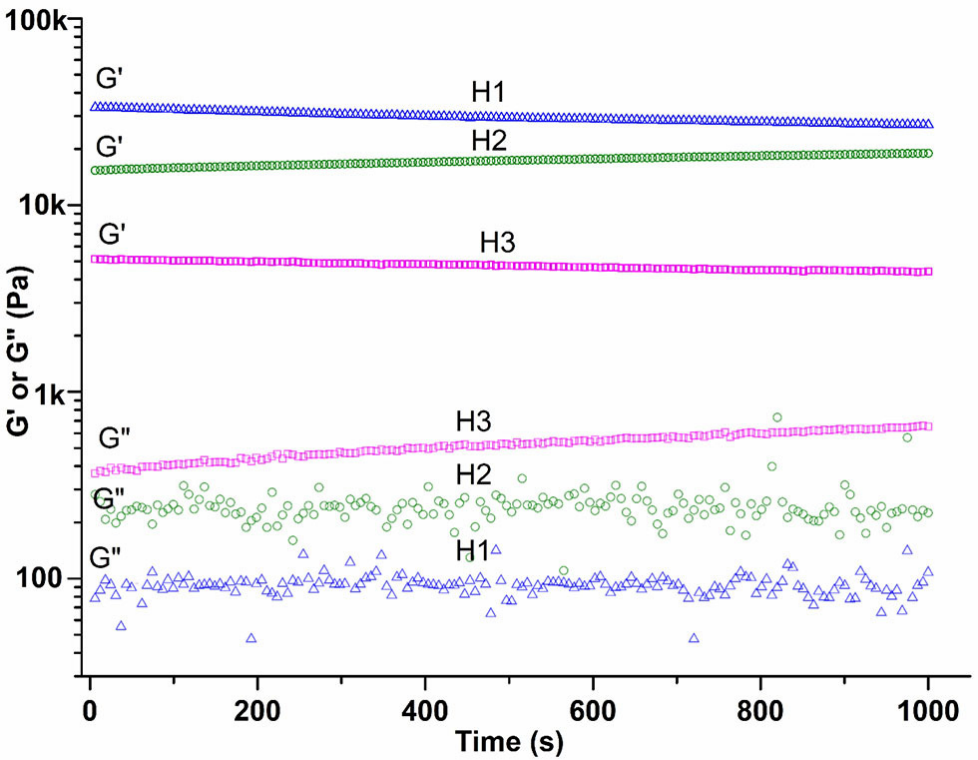
Frequency dependence of the dynamic storage modulus (G′) and the loss modulus (G″) of hydrogels with different polymer contents (H1, 20%; H2, 15%; H3, 10%).

To characterize the mechanical properties of the hydrogel, we monitored the change of storage modulus (G′), and loss modulus (G″) over time. As shown in Figure 9, the values of storage modulus show little frequency dependence and they are 33k, 15k, and 5k Pa, at the frequency of 10.0 rad/s for gel H1, gel H2, and gel H3, respectively, which suggests that gels with higher polymer contents are mechanically more stable than those with lower ones. The values of loss modulus (G″) are about 90 Pa, 240 Pa, and 410 Pa, at the frequency of 10.0 rad/s for gel H1, gel H2, and gel H3, respectively, which suggest that these gels have low viscous property and the viscous property was increased with increasing the water content of hydrogels.

## Conclusions

In conclusion, a poly(ethylene glycol) with a single cinnamaldehyde acetal unit in the polymer chain was synthesized successfully using a novel synthetic cinnamaldehyde acetal diethylene glycol (CADEG) as initiator. This HO-PEG(ca)-OH is non-toxic and would be degraded into a cinnamaldehyde and a PEG diol with around half of the average molecular weight of HO-PEG(ca)-OH in acid environment. A whole polyethylene glycol based hydrogel was easily fabricated by thiol-ene “click” reaction in alkalescence aqueous solution using acrylate-PEG(ca)-acrylate and 4-arm PEG-SH as raw materials at room temperature.

## Data Availability Statement

All datasets generated for this study are included in the article/Supplementary Material.

## Author Contributions

ZhiL, ZhoL, and XZ designed the studies. XZ conducted experiments with assistance from PS, DL, and PC for polymer synthesis and characterization, fabricating hydrogel, and cell culture. ZhiL and ZhoL wrote the manuscript. All coauthors read and approved the manuscript.

## Conflict of Interest

The authors declare that the research was conducted in the absence of any commercial or financial relationships that could be construed as a potential conflict of interest.
