# Supplementary material for: Poly(ethylene glycol)s With a Single Cinnamaldehyde Acetal Unit for Fabricating Acid-Degradable Hydrogel
Source: Front Chem. 2020 Sep 15;8:839. doi: 10.3389/fchem.2020.00839 (PMC7522333; doi:10.3389/fchem.2020.00839)
Supplement: Supplementary file 1 [file Table_1.DOCX]

Supporting Information

**Poly(ethylene glycol)s with a single cinnamaldehyde acetal unit for fabricating acid-degradable hydrogel**

**Xinyue Zhao^#1^, Pengfei Shan^#1,2^, Haiwei Liu^3^, Daai Li^1,2^, Peihan Cai^2^, Zhongyu Li*^2^, Zhihui Li*^1^**

^1^School of Optometry and Ophthalmology and Eye Hospital, State Key Laboratory of Optometry & Vision Science, Wenzhou Medical University, Wenzhou 325027, China

^2^College of Chemistry and Materials Engineering, Wenzhou University, Wenzhou 325027, China,

^3^The Department of Neurology, The First Affiliated Hospital of Wenzhou Medical University, Wenzhou, 325000, China

^#^These authors contributed equally.

**^*^ Correspondence:**

lizy@wzu.edu.cn (Z.Y. Li); smart_dream2010@yahoo.com.hk (Z.H. Li)

**The cytotoxicity of degradated polymer**

Fig. S1 NIH3T3 cells were subjected to 0.00001, 0.0001, 0.001, 0.01, 0.1, 1 mg/mL of degradated polymer (M_n_=1.16 × 10^3^ g/mol, by GPC) from hydrogel for 24, 48, 72 h before undertaking a MTS assay. The results are shown as percent viability compared to the control with media only. Each sample was replicated 6 times.
